# Supplementary figures and images for: Caught on camera: Field imagery reveals the unexpected importance of vertebrates for biological control of the banana weevil (Cosmopolites sordidus Col. Curculionidae)
Source: PLoS One. 2022 Sep 20;17(9):e0274223. doi: 10.1371/journal.pone.0274223 (PMC9488773; doi:10.1371/journal.pone.0274223)

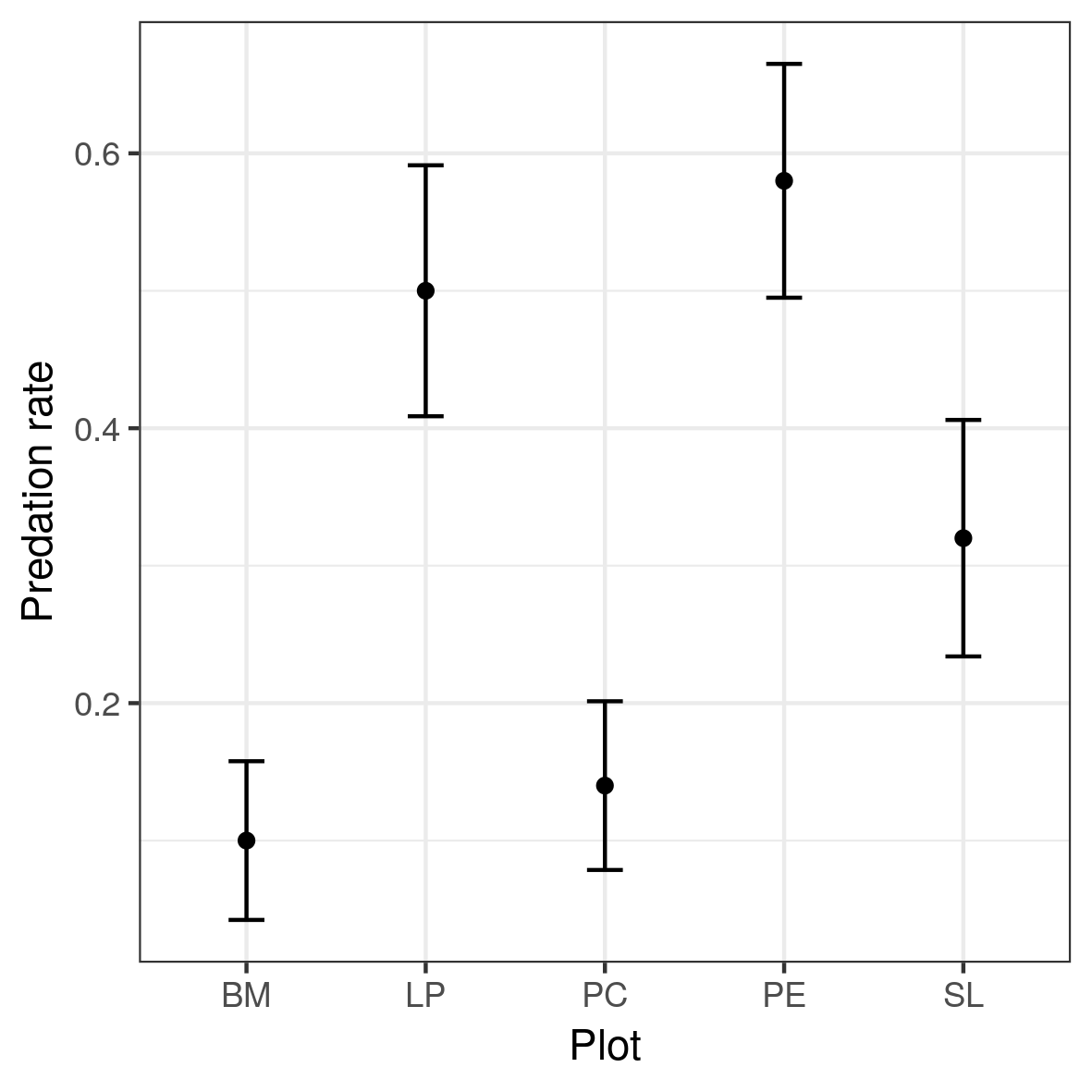

Supplement: S1 Fig — The sanitary ranking of plots was based on assessment of weevil infestation level before the experiment, damage reported by the farmer, and age of the plot; the ranking increased as the values of these variables increased. The PC plot was newly planted with in-vitro propagated banana plants and lacked any history of weevil infestation because the plot was not cultivated before, i.e., the absence of weevil damage in the PC plot was probably not caused by regulation by natural enemies. On the contrary, BM plot is planted with in-vitro propagated plants but banana was already cultivated before and the plot had an already installed weevil population. (TIF) [file pone.0274223.s001.tif]
