# Supplementary material for: Caught on camera: Field imagery reveals the unexpected importance of vertebrates for biological control of the banana weevil (Cosmopolites sordidus Col. Curculionidae)
Source: PLoS One. 2022 Sep 20;17(9):e0274223. doi: 10.1371/journal.pone.0274223 (PMC9488773; doi:10.1371/journal.pone.0274223)
Supplement: Attachment — Submitted filename: Minor Corrections.docx [file pone.0274223.s002.docx]

**Minor corrections and suggestions are below:**

Line 21: Should read “second” not “2^nd^”.

Line 34: Should read “67% of predation events” not “67.0% of the predation events”.

Line 37: I presume the time was taken specifically from image metadata? Would be good to specify.

Line 37: Should read “coincided” instead of “coincides”.

Line 41: I would say “Based on these results, we advocate” – it is the authors advocating this by interpreting the results.

Line 66: “specific information” or “important information” rather than just “information”

Line 69: Should read “biases such as” instead of “biases as”

Line 70: Should read “Without monitoring or use…” – the second without is unnecessary.

Line 71: No comma is needed after footnote (70).

Line 77: Should read “be biased” instead of “have biases”.

Line 86-87: I would start this sentence with “For example” to show you’re just giving one example use of automated image analysis.

Line 88: Should read “a prey animal” instead of just “a prey”.

Line 91: Should read “without the need for” instead of “without the need of”.

Line 98: “See” can be removed from references. It’s redundant to have both *e.g.* and see.

Line 104: While I understand you are trying to explain the term “corm” in layman’s terms, technically it is not the same as a bulb from a botanical point of view. I would maybe rephrase as “the storage organ at the base of the banana pseudostem” or similar.

Line 111: I would say “evidence of the role of vertebrates” is considered anecdotal.

Lines 117-118: In question 1 you refer to *C. sordidus* whereas in question 3 you refer to the “banana weevil”. I would be consistent and refer to it the same way in both questions.

Line 118: Should read “24-hour” instead of “24-h”.

Line 127: Should read “include” instead of “provide”.

Line 129: Should read “before or during” instead of “for”.

Line 131: Should read “ranged” instead of “range”.

Lines 136-138: You should be consistent in writing the number as either “two” or 2, not a mixture of both. A good rule of thumb is to write any numbers under 11 as words (*e.g.* ten, nine, eight). Either way, pick one method and be consistent throughout the text.

Line 147: See previous comment re “5”.

Line 174: Should read “24-hour” instead of “24-h”.

Line 204: Should read “where” instead of “were”.

Line 221: Should read “was counted” instead of “counted”.

Line 235: Should read “were detected” instead of “have been detected”.

Line 240: Should read “strongly suggested that the disappearance of the weevils was due to predation,”

Line 241: should read “disappearance” and “appearance” instead of “disappearing” and “appearing”.

Line 248-249: I would start the sentence with “Because of this, we chose to focus only on observed predator species,” for clarity.

Line 256-257: Should read “24-hour” instead of “24-h”.

Line 259: You state that *S. murinus* appears to be active only at the beginning of the night, however Fig. 4 shows S. murinus predation throughout the night, up until 04:00.

Lines 261-262: Since these are running totals, it would make more sense to say “had been consumed after” rather than “were consumed within”.

Line 293: Should read “had previously” instead of “has previously”.

Line 298: Should read “the range of possible management solutions” (or similar) rather than “the possible management solutions.”

Line 302: Should read “24-hour” instead of “24-h”.

Line 303: Should read “its prey” instead of “the prey”.

Line 321: Should read “eighth” instead of “8^th^”.

Line 322: the “la” in “La Réunion” should be capitalised.

Line 322: Should read “50% less” instead of “a 50% deficit in”.

Line 329: Should read “eggs **only** remain accessible for predation **for** a relatively short time”.

Line 331: Should read “difficult to access for” instead of “of difficult access by”.

Line 336-337: Should read “better regulation overall” instead of “a better regulation”.

Line 341: Should read “unconfirmed” instead of “unsuspected”.

Line 342: Should read “potential utility” instead of just “potential”.

Line 342: Should read “By using images” instead of “With images”.

Line 352: Can just read “some sequences” instead of “some image sequences” to avoid repetition.

Line 371: Should read “unknown” instead of “unsuspected”.

Line 383: Should read “range of possible management strategies” instead of just “possible management”.

Line 390: Should read “to” instead of “in order to”.

Line 391: Should read “near or within” instead of “near of within”.

Line 403: Should read “the key pest predator species” (or similar) instead of just “the key species”.

Line 408: Should just read “conceived” instead of “conceived of”.

Line 410: Possibly say “critical input” instead of “critically”.

Figures 4, 5 and 6: In general, figure captions should be able to be understood without reading through the entire text. In these graph captions you are missing some key information, namely 1) the prey species (banana weevil), 2) that these readings are from multiple sites 3) that these readings were taken over a 24-hour period.

Figure 5: Caption should read “species” not “specie”.

Figure 5: This graph is a little confusing because your caption and axis title claims to show the total number of predation events, but you also include “recovered” weevils, which clearly were not predated! So what your X-axis is should actually be titled is simply “Number of weevils”. You could then rename the caption something like “Number of predated vs. unpredated banana weevils per plot over 24 h, coloured by predator species”. As I mentioned before, the 24-hour time frame and prey species are important information to include.
